# Litter removal reduced soil nitrogen mineralization in repeated freeze-thaw cycles

**DOI:** 10.1038/s41598-018-38431-4

**Published:** 2019-02-14

**Authors:** Yulian Yang, Li Zhang, Xinyu Wei, Ya Chen, Wanqin Yang, Bo Tan, Kai Yue, Xiangyin Ni, Fuzhong Wu

**Affiliations:** 1 Long-Term Research Station of Alpine Forest Ecosystems, Key Laboratory of Ecological Forestry Engineering, Institute of Ecology and Forestry, Sichuan Agricultural University, Chengdu, 611130 China; 2 Ecological Security and Protection Key Laboratory of Sichuan Province, Mianyang Normal University, Mianyang, 621000 China

## Abstract

Repeated freeze-thaw cycles (FTCs) can alter the relationships between plant litter and soil nitrogen (N) mineralization in subalpine ecosystems, but little information is available about the underlying mechanisms. Therefore, a controlled soil incubation experiment was carried out to study the effects of litter removal on soil N mineralization during FTCs, and the results indicated that FTCs promoted soil N mineralization more than the continuously frozen or nonfrozen condition did. Litter removal promoted soil ammonium N (NH_4_^+^-N) and dissolved organic N (DON) as well as the cumulative N mineralization (CNM) and ammonification, but it reduced the soil microbial biomass N (MBN) in the early stage of FTCs. With an increasing number of FTCs, litter removal significantly reduced the CNM but increased the soil MBN. The modified first-order kinetics model was verified under incubation conditions and predicted a lower soil N mineralization rate in FTCs with litter removal. In addition, the dominant factor impacting soil N mineralization was soil NO_3_^−^-N, and soil MBN had a greater influence on soil N mineralization when litter remained than when it was removed. These results further clarify the mechanism driving the effect of plant residues on soil N cycling.

## Introduction

Soil nitrogen (N) mineralization, which regulates N availability and supply, is a key process in forest ecosystems^1–4^. Although soil N is mainly formed through the partial decomposition and transformation of plant residues^3,5,6^, the rate of N transformation in the soil is closely linked to litter quality^7–9^ and climate factors^10–12^. Previous studies have shown that carbon (C) and nutrients from the decomposition of plant residues can provide adequate energy and nutrients for the growth of soil microorganisms^13,14^ and that increased microorganism quantity and activity^15^ accelerate soil N mineralization. Furthermore, microorganisms can improve N availability by immobilizing mineralized mineral N within their cells^14^ and can even retain most immobilized organic N in N-limited soil environments, both of which result in low N mineralization^16^. In addition, altered microbial community compositions in response to plant residue input^14,17^ may clearly affect microbial N-use efficiency^18^; in turn, these alterations affect the N mineralization process. However, the relationship between litter and soil N mineralization is unclear, so knowledge of how plant residue inputs affect both the soil N mineralization process and its influencing factors could significantly contribute to our understanding of nutrient cycling, especially that of N in forest ecosystems.

Freeze-thaw cycles (FTCs) in soils are common phenomenon in high-altitude and high-latitude regions as well as some temperate zones^10,11^, and they may affect soil N mineralization when litter is deposited in subalpine forests. FTCs break down soil aggregates, thereby increasing the availability of soil N to soil microorganisms via exchange across the resulting increased surface area^11^, and repeated FTCs can promote microorganism cell destruction and stimulate the metabolism of surviving microorganisms via their internal release of C and N in the soil^10,11,19,20^. Mechanical fragmentation also stimulates the release of C and nutrients from decomposing plant residue^13,21–23^, thereby increasing microbial activity and N immobilization^9^. Additionally, although a relatively high fungi/bacteria ratio has been reported when litter is input into a system^17,24^, substantially reduced amounts of fungi and stable amounts of bacteria due to FTC^25^ may impact soil microorganism community structure, affect microbial N-use efficiency, and further alter organic N decomposition and immobilization processes^23^.

Ongoing global climate warming has been altering the intensity, frequency and duration of FTCs through milder winters and thinner snow cover^10,26^, which may strongly affect soil structure, soil microorganism activity and soil microorganism communities^27–29^ and therefore alter the relationship between litter and soil N mineralization. To date, the individual effects of plant residue inputs and FTCs as well as their comprehensive effects on soil N mineralization are uncertain and urgently need to be elucidated. We hypothesized that litter removal would reduce soil N mineralization under repeated FTCs because more C and nutrient sources would be available from plant residue decomposition, although the soil N pools would differ with various FTC frequencies.

To test this hypothesis, a controlled soil incubation experiment to simulate freeze-thaw conditions was conducted using subalpine forest soils collected from a typical subalpine forest in the upper reaches of the Yangtze River, China. Soil N pool and N flux dynamics were determined and compared to understand the response of soil N mineralization to litter and freeze-thaw patterns. We specifically focused on the following research questions: (1) Do soil N pools and N fluxes decrease in response to litter removal under FTC conditions? (2) How do FTC regimens (e.g., frequency and duration) influence N pools and fluxes when plant residues are removed? The answers to these questions will provide basic data on the effects of the interaction between litter and FTCs on soil N cycling in subalpine forest ecosystems and will further help to clarify the underlying mechanism.

## Results

### Soil N pool dynamics

**Figure 1:**
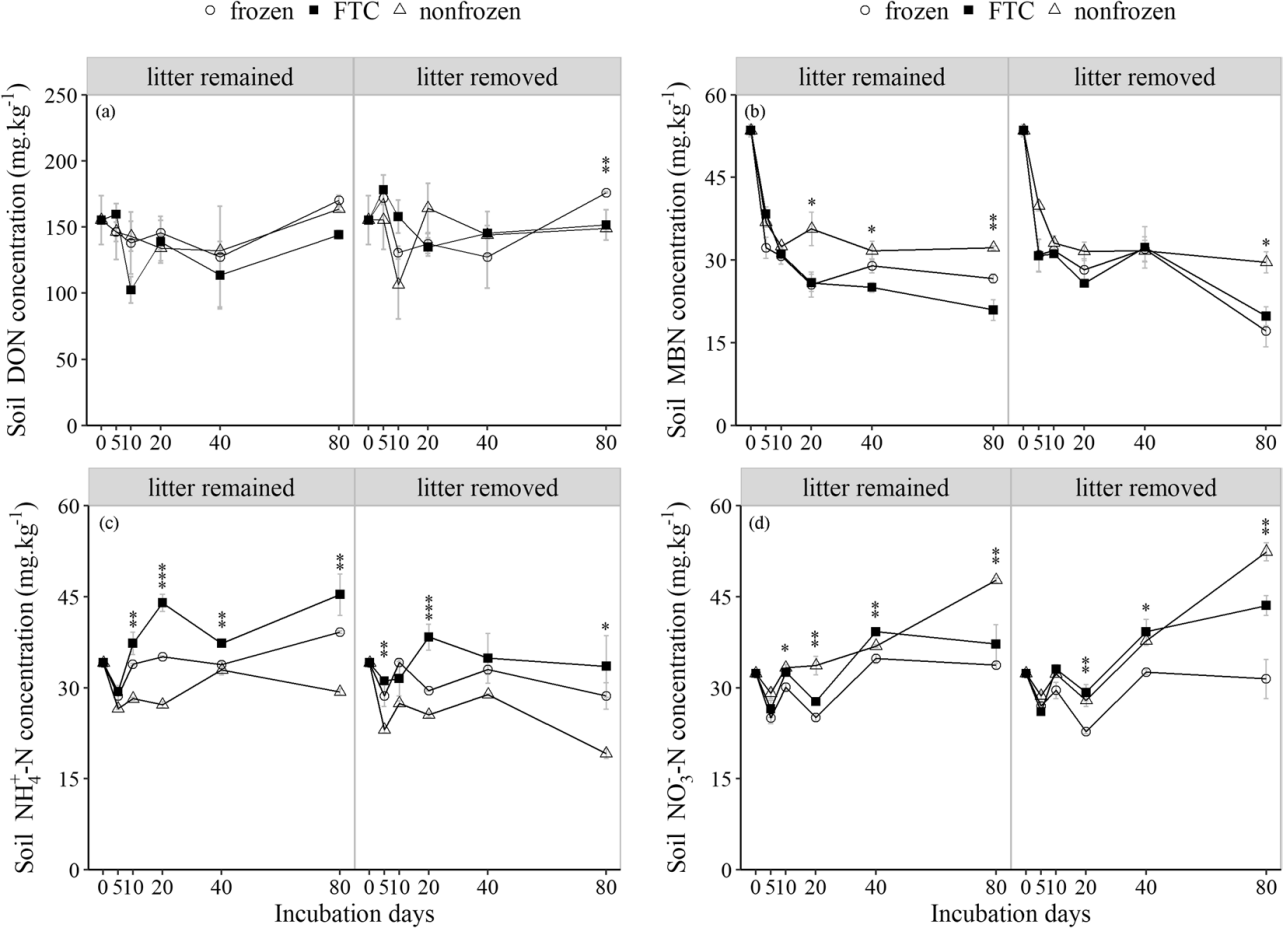
The dynamics of soil nitrogen (N) pools varied with litter and freeze-thaw treatments (mean ± SE, n = 3). *Denotes a significant difference between different freeze-thaw treatments for the same litter treatments and incubation days. *Indicates *P* < 0.05; **indicates *P* < 0.01; and ***indicates *P* < 0.001.

The effects of litter removal on soil N pools clearly differed due to freeze-thaw patterns and incubation time (Fig. 1, Table 1). Compared with the frozen and nonfrozen treatments, the FTC treatment significantly increased the soil dissolved organic N (DON) by 54.02% and 27.93% after 10 and 40 days of incubation, respectively, when the litter was removed (Fig. 1a). The soil microbial biomass N (MBN) concentrations were lower in the FTC and frozen treatments than in the nonfrozen treatment between 10 and 80 days in the two litter treatments, and the greatest declines were 34.99% and 42.20% in the litter-remained and litter-removal treatments, respectively. In addition, compared with the frozen and nonfrozen treatments, the FTC treatment significantly reduced the soil MBN concentration by 19.7% after 5 days of incubation, whereas the soil MBN increased by 28.94% after 40 days of incubation when the litter was removed (Fig. 1b).

**Table 1:** Repeated measures ANOVA results for the responses of soil nitrogen (N) pools and fluxes to freeze-thaw treatments (FT), litter treatments (L), incubation days (D) and their interactions. *P < 0.05; **P < 0.01; ***P < 0.001.

Factor	DON	MBN	NH_4_^+^-N	NO_3_^−^-N	CA	CN	CNM
FT	2.66	182.10^***^	24.96^**^	172.8^***^	24.96^**^	172.8^***^	16.11^*^
L	20.16^*^	1.27	48.37^***^	0.05	48.37^***^	0.05	22.45^*^
D	8.36^**^	51.30^***^	16.17^**^	183.50^***^	16.17^**^	183.50^***^	69.45^***^
FT × L	2.72	1.44	1.39	14.41^*^	1.39	14.41^*^	0.77
FT × D	0.81	11.01^***^	8.70^**^	57.06^***^	8.70^**^	57.06^***^	8.24^***^
L × D	3.07	9.63^**^	46.65^***^	10.64^**^	46.65^***^	10.64^**^	8.28^**^
FT × L × D	8.25^***^	9.16^***^	4.78^**^	4.04^**^	4.78^**^	4.04^**^	2.18

Regardless of whether the litter remained or was removed, compared with the nonfrozen treatment, the FTC and frozen treatments significantly increased the soil ammonium N (NH_4_^+^-N) level by 10.54–74.82% and 0.03–49.41%, respectively. Soil NH_4_^+^-N in the FTC and frozen treatments increased in response to litter removal after 5 days of incubation, but during incubation periods of 10 to 80 days, litter removal reduced the soil NH_4_^+^-N, with the greatest declines of 26.09%, 26.83%, and 34.58% occurring in the FTC, frozen, and nonfrozen treatments, respectively, after 80 days of incubation (Fig. 1c). The soil nitrate N (NO_3_^−^-N) concentrations differed slightly among all the treatments, and the levels were lower in the frozen treatment than in the FTC or nonfrozen treatment during incubation periods of 10 to 80 days. Compared with that in the frozen or nonfrozen treatment, litter removal in the FTC treatment increased soil NO_3_^−^-N by 5.45% and 17.10% after 20 and 80 days of incubation, respectively (Fig. 1d).

### Soil N flux dynamics

**Figure 2:**
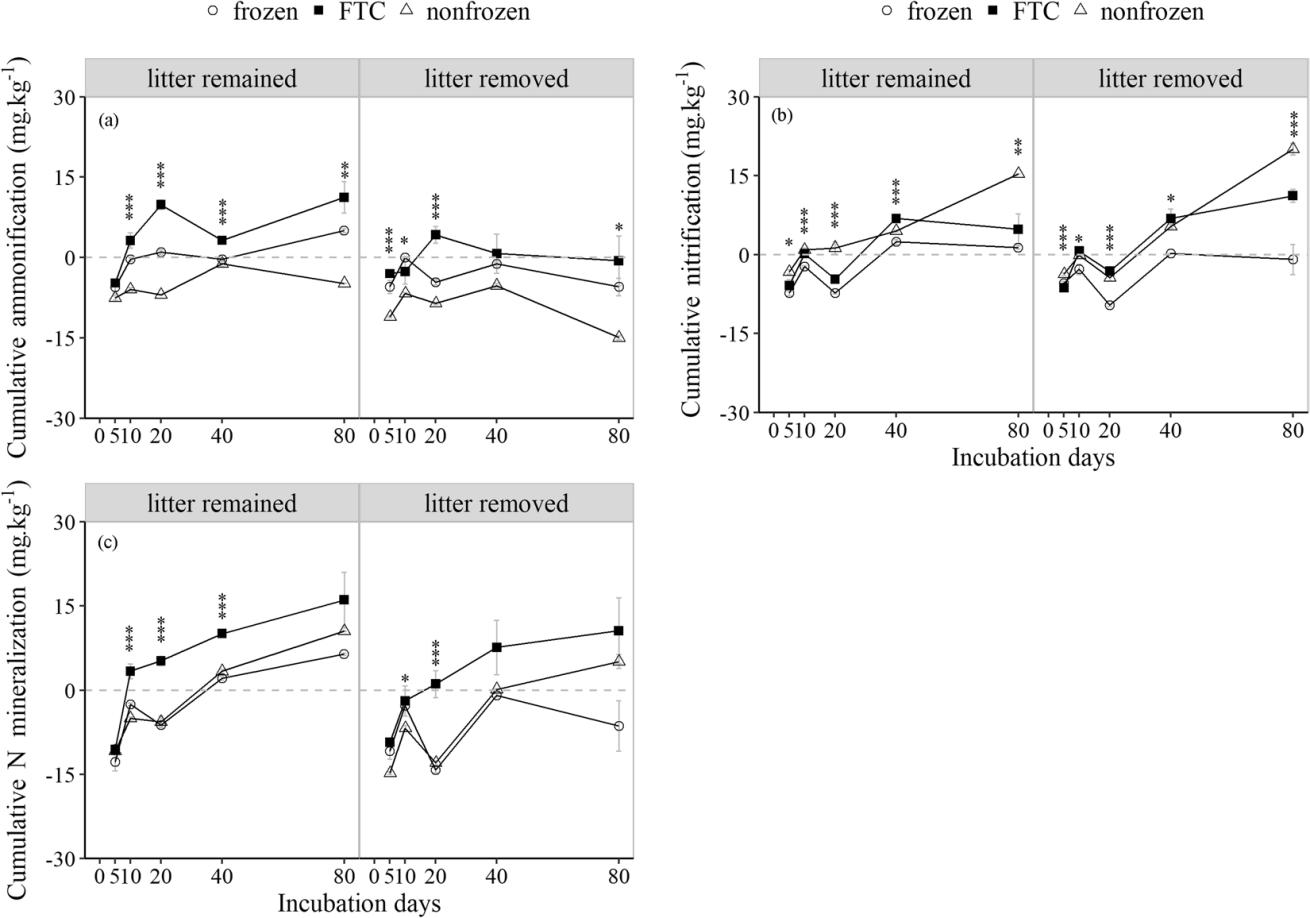
The soil cumulative ammonification (CA), cumulative nitrification (CN) and cumulative N mineralization (CNM) dynamics varied with litter and freeze-thaw treatments (mean ± SE, n = 3). *Denotes a significant difference between different freeze-thaw treatments for the same litter treatments and incubation days. *P < 0.05; **P < 0.01; ***P < 0.001.

Litter treatment, freeze-thaw treatment and their interactions had significant effects on soil N fluxes, but there were differences among different culture periods (Fig. 2, Table 1). In general, the soil cumulative N mineralization (CNM) and cumulative nitrification (CN) in the FTC treatment increased as the incubation time increased, whereas the soil cumulative ammonification (CA) exhibited only a slight ascending tendency. Unlike the soil CNM in the FTC treatment, which exhibited a period of rapid increase in the early culture stage and then increased slowly, there was a downward trend in the frozen and nonfrozen treatments after 20 days of incubation (Fig. 2c). After 5 days of incubation, the soil CNM, CA, and CN values were all negative, and the N cycle was dominated by N immobilization. During the incubation period from 10 to 80 days, litter removal inhibited the soil CNM, and the AR increased in all treatments, although the FTC treatment promoted the soil CNM and AR. Most of the soil NMR, NR and AR values in the litter-remained treatment were positive, and the N cycle was dominated by N mineralization. Moreover, the duration required for the soil CNM and CA values to become positive was extended by litter removal in the FTC treatment (Fig. 2a,c).

### Soil N mineralization model

The best-fit modified first-order kinetics model obtained in the nonfrozen and litter-remained treatment emphasized the results that soil N mineralization progress was influenced by litter removal and soil freeze-thaw cycles (Table 2). Compared with the FTC and nonfrozen treatments, the frozen treatment reduced the contribution of the soil MBN to soil N mineralization with the decreased values of MBN (N_e_) when litter remained, whereas the model was not fit when litter was removed because of a greater number of uncertainty factors. The FTC treatment significant increased the k values, which were 66.67% and 77.78% higher than those of the frozen and nonfrozen treatments, respectively, when litter remained and 80.00% higher than those of the nonfrozen treatment, with litter removal. Furthermore, the k value was higher when litter remained than when it was removed, especially in the FTC treatment.

**Table 2:** Parameters of the modified first-order kinetics model of nitrogen (N) mineralization in the different treatments.

Treatment	Parameter			AIC	F	*P*
	N_0_ (mg kg^−1^)	N_e_ (mg kg^−1^)	k (day^−1^)			
Litter remained						
FTC	−21.5^A^	13.41^A^	0.09^B^	56.29	19.72	<0.001
Frozen	−13.04^A^	8.29^A^	0.03^A^	45.29	18.6	<0.001
Nonfrozen	−12.07^A^	20.28^A^	0.02^A^	28.54	75.52	<0.001
Litter removed						
FTC	−14.07^A^	10.73^A^	0.05^AB^	62.75	6.96	<0.01
Frozen	not fit	not fit	not fit	not fit	not fit	not fit
Nonfrozen	−15.28^A^	16.84^A^	0.01^A^	49.01	27.28	<0.001

The parameter values followed by the same capital letter are not significantly different between the five treatments (*P* > 0.05).

## Discussion

In this study, different FTC regimens clearly affected soil N cycling in the subalpine forest, which is consistent with the results of previous studies^10,30–32^, in which FTCs increased soil inorganic N concentrations and promoted soil N mineralization^10,11^. Moreover, our hypotheses that litter removal would reduce the soil CNM and slow soil N mineralization and that these effects would be even greater under the FTC or frozen treatment were well supported in the present study. However, the soil DON and MBN tended to increase in response to litter removal in the FTC treatment. An understanding of soil N mineralization processes both when litter is removed and when the soil is subjected to FTCs is essential for explaining N cycling in alpine forests.

The size of the soil organic N pool depends on the balance between the formations of soil organic N from the decomposition of plant litter and its mineralization into inorganic N by soil organisms^33^. Increases or decreases in the abundance of soil microorganisms, the drivers of N cycling, can directly affect the soil N pools and N fluxes in alpine forests^10^, and the number of microorganisms present can rapidly decrease due to partial microbial resistance to FTCs or freezing stress^11,20,28^. Therefore, in this study, extremely adverse environmental conditions in conjunction with no additional energy or nutrient inputs caused the soil MBN to decrease when litter was removed directly (Fig. 1b). Although nutrients from disrupted microbial cells would promote soil DON, NH_4_^+^-N and NO_3_^−^-N^10^, lower microorganism activity due to limited energy and nutrients would account for the increased soil DON and NH_4_^+^-N when the litter was removed (Fig. 1a,c). There are two reasonable explanations for the increased MBN in response to litter removal as the number of FTCs and the continuous acquisition of nutrients in the soil increased in the FTC treatment: (1) fewer N resources increase soil MBN because microorganisms retain most of the immobilized organic N in their cells^16^, and (2) the affected groups of microorganisms also increase the soil MBN. On the one hand, substantially reduced amounts of fungi due to FTCs^25^ and the relatively lower fungal demand for N may directly cause MBN to decrease. On the other hand, relatively high amounts of fungi when litter remains^18,24^ will lead to a decrease in soil MBN. In contrast, litter removal reduced the soil NH_4_^+^-N in the FTC treatment, although the FTCs generally increased the soil NH_4_^+^-N, which was mainly attributable to soil microorganisms. The soil CNM and CA showed trends opposite to that of the soil MBN concentration due to regulation by microorganisms (Figs 1,2). As concurrent processes, higher microorganism N immobilization means lower soil N mineralization, thereby reducing soil NH_4_^+^-N levels. The significant negative relationship between soil CNM and MBN (Table 3) and the reduced soil CNM and SA (Fig. 2) in response to litter removal also verified these results. However, the effects on soil SA and SN were not consistent (Fig. 2), which was mainly due to the preference of the microorganisms to use NH_4_^+^-N rather than NO3^−^-N^3^. However, heterotrophs could no longer immobilize NH_4_^+^-N, but soil nitrifiers could still oxidize NH_4_^+^-N into NO_3_^−^-N. Therefore, NO_3_^−^-N could accumulate when C substrates were unavailable^34^, especially during the later culture period, in response to litter removal in the absence of sufficient C. In addition, the reduced soil CNM and CA and the inhibition of soil N mineralization by litter removal in the FTC treatment (Fig. 2) indicated the importance of litter to soil N mineralization and N cycling.

**Table 3:** Regression model of soil cumulative nitrogen (N) mineralization and factors under different treatments. *P < 0.05; **P < 0.01; ***P < 0.001.

Treatment	Best regression model					AIC	R^2^_adj._	*P*
	a_0_	a_1_X_1_	a_2_X_2_	a_3_X_3_	a_4_X_4_			
Litter remained								
FTC	y = −41.27^***^	0.82 (NO_3_^−^-N)^***^	0.76 (NH_4_^+^-N)^***^	−0.30 (MBN)^***^	−0.01 (DON)^*^	−16.94	0.997	<0.001
Frozen	y = −53.60^***^	1.00 (NO_3_^−^-N)^***^	0.89 (NH_4_^+^-N)^***^	−0.23 (MBN)^*^	−0.02 (DON)	1.10	0.983	<0.001
Nonfrozen	y = −46.50^***^	1.05 (NO_3_^−^-N)^***^	0.70 (NH_4_^+^-N)^***^	−0.20 (MBN)^*^	−0.04 (DON)^*^	−1.15	0.988	<0.001
Litter removed								
FTC	y = −50.83^***^	0.95 (NO_3_^−^-N)^***^	0.79 (NH_4_^+^-N)^***^	−0.04 (MBN)	−0.04 (DON)^**^	−8.05	0.993	<0.001
Frozen	y = −50.58^***^	1.06 (NO_3_^−^-N)^***^	0.61 (NH_4_^+^-N)^***^	−0.04 (DON)^**^		−0.06	0.973	<0.001
Nonfrozen	y = −48.46^***^	0.90 (NO_3_^−^-N)^***^	0.80 (NH_4_^+^-N)^***^	−0.19 (MBN)^**^	−0.02 (DON)^***^	−3.70	0.990	<0.001

The progress of soil N mineralization and its influencing factors are very complex, and based on previous studies, it is more likely that there are at least two types of organic N sources with different mineralization potentials in the soil mineralized N pools^35,36^, as has been verified in alternating dry and wet environments^37^. Our study reached a similar conclusion, and the higher values of k indicate faster soil N mineralization rate; in addition, the negative values of N_0_ may due to the soil N mineralization-immobilization cycle process, which needs to be studied further to reveal the N mechanism model. A previous study considered N_e_ a small source of easily mineralized N that would be exhausted within a week^37^; however, the little differences from N_0_ in our results are possibly due to the adaptation of soil microorganisms to the freeze-thaw environment of alpine forests. Moreover, our regression results also verified soil MBN as a significant factor influencing soil CNM when litter remained (Table 3). In contrast, soil microbial activity may be altered little when there is no extra energy or nutrient input to the soil environment under the disturbance of the soil environment by FTC_S_, so soil MBN did not significantly affect soil CNM in the FTC and frozen treatments (Table 3). In a word, differences in key impact indicators between the litter-remained and litter-removal treatments also indicate different mechanisms of soil N conversion (Tables 2 and 3). Moreover, reduced amounts of soil microbes would decrease the gross mineralization under FTC conditions, but decreased immobilization of NH_4_^+^-N and NO_3_^−^-N by soil microorganisms and the adaptation of microorganisms to long-term FTCs would counterbalance these effects^10^, causing the interactions among freeze-thaw patterns, litter treatment and incubation time to have no significant impact on the soil CNM (Table 1).

## Conclusions

In summary, altered FTC regimens due to ongoing global climate can change the relationships between plant litter and soil N mineralization in subalpine ecosystems, but these changes are controlled by the frequency or duration of the FTCs. In the early stage of FTCs, litter removal increased soil NH_4_^+^-N, DON, CNM and CA but reduced soil MBN. In contrast, litter removal decreased soil NH_4_^+^-N, NO_3_^−^-N, CNM and CA but increased soil MBN and the CN in the later stage. In conjunction with remaining litter, the FTCs clearly promoted the soil CNM. In addition, the most important factor that impacted soil N mineralization was soil NO_3_^−^-N, and soil MBN influenced soil N mineralization more strongly when the litter remained than when it was removed. These findings confirmed that plant residue inputs promote N mineralization under frequent FTCs, and the results of this study will facilitate the elucidation of the mechanism underlying the effects of plant residues on soil N cycles.

## Materials and Methods

### Experimental design

In May 2017, samples of both soils from the organic layer (0–15 cm) and undecomposed litter on the soil surface were randomly collected from three *Picea purpurea* forest sites with similar elevations and age structures. The forests are located in the Wanglang National Nature Reserve, Sichuan Province, China (103°55′–104°10′ E, 32°49′–33°02′ N) on the eastern Qinghai-Tibetan Plateau at an altitude of 2300–4980 m, and the freeze-thaw season generally extends from late October to late April of the following year. The mean annual temperature ranges from 2.5 to 2.9 °C, and the maximum and minimum monthly means are 12.7 °C and −6.1 °C in July and January, respectively^38^. The annual precipitation ranges from 801 to 825 mm depending on the elevation, with most falling between May and August^39^. The dominant trees are *P. purpurea*, *Abies faxoniana*, *Sabina saltuaria*, *Betula albosinensis*, and *Betula utilis*, and the dominant shrubs are *Salix cupularis*, *Fargesia denudata* and *Elaeagnus pungens*. The soil type of the *P. purpurea* forest is a dark brown forest soil according to the Chinese soil genetic classification^40^ and is classified as a type of Cambisol in other classification systems^41^. The physical and chemical properties of the soil are listed in Table 4.

**Table 4:** Basic physical and chemical properties of the soil and litter (mean ± SE, n = 3).

	OC (g kg^−1^)	TN (g kg^−1^)	TP (g kg^−1^)	C/N	C/P	WHC (kg kg^−1^)	B (g cm^−3^)	pH
Soil	57.77 ± 2.63	3.88 ± 0.16	1.18 ± 0.04	14.92 ± 1.23	48.96 ± 3.70	1.23 ± 0.02	0.74 ± 0.11	5.42 ± 0.05
Litter	348.00 ± 6.10	2.23 ± 0.03	0.76 ± 0.02	155.79 ± 5.03	456.76 ± 14.37	/	/	/

OC, Organic carbon; TN, Total nitrogen; TP, Total phosphorus; WHC, Water-holding capacity; BD, Bulk density.

After they were passed through a 2-mm sieve to remove roots and gravel, well-mixed soil samples (250 g) were placed into 350-ml gas-permeable polyethylene tanks, brought to a surface area of 50.24 cm^2^ and wetted to 60% of their water-holding capacity. The samples were then incubated at 5 °C for three days to increase the tendency of the soil to remain stable after disturbance. The leaf litter was air-dried for two weeks at room temperature. A full factorial design was used to test the effects of different freeze-thaw patterns (FTC, frozen and nonfrozen) and litter (remained vs. removed) on soil N mineralization. There were three temperature settings in triplicate that were controlled by different freezers and based on previously reported observational data and temperature dynamic characteristics^21^: one set was subjected to 80 FTCs, where each FTC involved 12 h at −5 °C and 12 h at 5 °C for a total of 24 h for each FTC; the other two sets were incubated at constant temperatures of −5 °C (frozen) or 5 °C (nonfrozen). The litter-removal treatment involved only of soil sampling in the culture tanks; the other subsample was subjected to the litter-remained treatment, in which 1.63 g of air-dried leaf litter was added to the soil surface in accordance with the annual leaf production in *P. purpurea* forests^39,42^ and the surface area of the culture tank.

The culture tanks were weighed twice weekly to determine any weight loss (assumed to be due to water loss), and distilled water was sprayed evenly over the surface of the soils to compensate for any differences^43^. Duplicate tanks were destructively sampled after 0, 5, 10, 20, 40, and 80 days; both the soil and leaf litter from the three culture tanks in the same freezer were mixed separately and then analyzed.

### Chemical and physical analyses

The soil pH was determined using a 1/2.5 soil/water mixture, and the soil bulk density and the water-holding capacity were determined by coring^44^. Soil and litter organic carbon (C) concentrations were determined using the dichromate oxidation-sulfate-ferrous titration method; soil and litter total N concentrations were determined by the macro-Kjeldahl method; and soil and litter total phosphorus (P) concentrations were determined by the acid melt-molybdenum stibium anticolor method^44,45^. The soil MBN was extracted with 0.5 mol L^−1^ potassium sulfate (K_2_SO_4_), and the concentration was determined by the chloroform fumigation extraction method^46^. The soil inorganic N and dissolved total N (DTN) were extracted with 2 mol L^−1^ potassium chloride (KCl), and the NH_4_^+^-N concentration in the soil was measured colorimetrically by the indophenol method^28^. The soil NO_3_^−^-N concentration was determined by the dual-wavelength colorimetric method^47^, and the soil DTN concentration was measured via potassium persulfate (KPS) oxidation-UV spectrophotometry^48^.

The soil DON concentration was determined by the differences between the soil DTN and inorganic N (sum of NH_4_^+^-N and NO_3_^−^-N)^49^. We define mineralizable N as the increase in inorganic N (NH_4_^+^-N and NO_3_^−^-N) during 80 days of incubation.

### Kinetic models for net N mineralization

The modified first-order exponential model (two-pool model) is expressed in the following form:

$${{\rm{N}}}_{{\rm{m}}}={{\rm{N}}}_{{\rm{e}}}(1-\exp (-{\rm{kt}}))+{{\rm{N}}}_{0}(\exp (-{\rm{kt}}))$$

where N_m_ represents the cumulative N mineralization (CNM, mg of N kg^−1^ soil); N_e_, the microbial biomass N (mg of N kg^−1^ soil); N_0_, the potentially mineralizable N (mg of N kg^−1^ soil); and k, the mineralization constant (or mineralization), which is expressed in days^−1^ because the incubation time (t) is expressed in days.

### Calculations and statistical analysis

Soil N pools and N fluxes under different freeze-thaw patterns in the same litter treatment during the same incubation period were calculated using one-way analysis of variance (ANOVA). Three-way repeated measures ANOVA on all three factors test was used to evaluate the effects of the freeze-thaw treatment, litter treatment, incubation duration and their interactions on the indices of the soil N pools and N fluxes. On the basis of the fitting analyses of the different N mineralization models^50^, three models, including the single first-order exponential model^51^, the modified first-order exponential model (two-pool model)^37^ and the mixed first and zero-order model^52^, were established to test N mineralization in the soils throughout the incubation period. Furthermore, the modified first-order exponential model (two-pool model) was selected to further obtain the mineralization parameter among treatments according to the lowest finite Akaike information criterion (AIC). Moreover, Tukey’s test was performed to evaluate the parameters among treatments. In addition, stepwise regression analysis was conducted to examine the main soil N pool factors that determine the soil CNM under different treatments, and the best general linear model was developed according to the lowest finite AIC. R software (version 3.4.3) was used to construct point plots and to perform ANOVAs, Tukey’s tests and stepwise regressions, and exponential regression and model selection were constructed and performed using Origin (version 9.0), respectively.
